# SmartVeh: Secure and Efficient Message Access Control and Authentication for Vehicular Cloud Computing

**DOI:** 10.3390/s18020666

**Published:** 2018-02-24

**Authors:** Qinlong Huang, Yixian Yang, Yuxiang Shi

**Affiliations:** 1 School of Cyberspace Security, Beijing University of Posts and Telecommunications, Beijing 100876, China; yxyang@bupt.edu.cn; 2 School of Science, Beijing University of Posts and Telecommunications, Beijing 100876, China; shiyx@bupt.edu.cn

**Keywords:** vehicular cloud computing, message access control, attribute-based encryption, message authentication, attribute-based signature

## Abstract

With the growing number of vehicles and popularity of various services in vehicular cloud computing (VCC), message exchanging among vehicles under traffic conditions and in emergency situations is one of the most pressing demands, and has attracted significant attention. However, it is an important challenge to authenticate the legitimate sources of broadcast messages and achieve fine-grained message access control. In this work, we propose SmartVeh, a secure and efficient message access control and authentication scheme in VCC. A hierarchical, attribute-based encryption technique is utilized to achieve fine-grained and flexible message sharing, which ensures that vehicles whose persistent or dynamic attributes satisfy the access policies can access the broadcast message with equipped on-board units (OBUs). Message authentication is enforced by integrating an attribute-based signature, which achieves message authentication and maintains the anonymity of the vehicles. In order to reduce the computations of the OBUs in the vehicles, we outsource the heavy computations of encryption, decryption and signing to a cloud server and road-side units. The theoretical analysis and simulation results reveal that our secure and efficient scheme is suitable for VCC.

## 1. Introduction

Vehicular cloud computing (VCC) is an emerging and promising approach to exploit the latest advances in sensing, the Internet of Things, wireless communications, and cloud computing technologies for future transportation [1,2], which may improve road safety and satisfy emerging service demands through message broadcasting. VCC typically consists of road side units (RSUs) and on-board units (OBUs). Particularly, VCC is regarded as an important development that interconnects people, vehicles and information, since numerous services based on vehicle systems may require cooperation among vehicles and RSUs. In order to maximize the overall communication and computation efficiency in VCCs, adaptive resource management has been proposed to provide hard quality of service guarantees in some recent studies [3,4]. That means, with the wireless and sensor network, the driver can enjoy various services in-vehicle based on VCC. The wide application of VCC depends on an efficient mechanism to ensure secure and effective message sharing, which is critical to enable emerging services.

Specifically speaking, let us consider the following practical VCC scenarios [5,6]. Regarding the social aspect, for instance, drivers in vehicles are often glad to share their experiences and traffic information with others who are on the same journey, and may also wish to discuss common interests with friends. Regarding the safety aspect, if there is an emergency (such as a traffic accident or a pavement collapse) on a certain road, the passing drivers may broadcast a warning message to nearby vehicles. If this message can be shared among vehicles in a short time, more serious traffic jams or serious accidents can possibly be prevented. The passing driver may also want to notify a police car and ambulance which is near the affected areas to deal with incidents at the same time. Therefore, it is important to provide efficient access control methods in VCC to guarantee reasonable message access. Unfortunately, adversaries may easily inject false information into the communication network, or even broadcast forged messages to the transportation system; unexpected situations may be caused by these security issues. Hence, message confidentiality, message authentication and access control are the most important problems that affect the VCC services [6]. In order to solve these security issues, traditional encryption mechanisms might be unsuitable.

The attribute-based encryption (ABE) is a cryptographic technique which provides fine-grained access control for encrypted data [7]. In particular, the ciphertext in ciphertext-policy ABE (CP-ABE) scheme can be decrypted only if the attribute set associated with a secret key satisfies the access policy. Hence, a receiver needs to own enough attributes to decrypt the broadcast message [8]. With this technique, both message confidentiality and access control are ensured in VCC. However, applying ABE in VCC has several challenges. Firstly, it brings a heavy key management burden to attribute authority (AA). The attributes of vehicles can be divided into two types in VCC [9], persistent attributes, for which the values remain constant, such as vehicle type and brand, and dynamic attributes, for which the values change frequently, such as road, direction and location. Hence, AA has to renew both the persistent and dynamic attribute keys of the driving vehicle when any dynamic attribute changes to guarantee the decryption capability of vehicles, which brings extra computation and communication overhead. Secondly, ABE introduces heavy computational overhead in data encryption and decryption phases, and this presents a serious challenge for resource-limited OBUs [10].

To ensure the origin of a message, message authentication schemes based on identity based signature (IBS) and attribute-based signature (ABS) in VCC have been studied. However, an IBS scheme would disclose the identification of the signer, which is undesirable. In an ABS scheme, the signer can generate a signature with his attributes issued by AA. Then, from the signature, the recipient vehicle can verify the signature by checking that the sender’s attributes satisfy the complex predicate policy without exposing the identity of the sender [11]. However, ABS also brings high computation costs, which cannot be adopted by OBUs directly.

In summary, it is important to maintain secure and reliable message broadcasting with low computation in VCC. In this work, we propose a secure and efficient message access control and authentication scheme for VCC, called SmartVeh, which features the following achievements.

(1) We provide a secure message access control framework in VCC based on hierarchical ABE (HABE). The framework consists of a trusted authority (TA), and a group of AAs which request secret parameters from the TA and generate persistent attribute keys or dynamic attribute keys for vehicles independently. Thus, vehicles can share confidential messages with other vehicles which satisfy the pre-defined access policy.
(2) We utilize ABS to enforce message authentication, which can authenticate messages by verifying whether the signer’s attributes satisfy the predicate policy. It also ensures message integrity by checking and maintaining the anonymity of vehicles.
(3) We present a secure outsourcing construction in VCC by delegating the heavy computations from resource-limited OBUs to the cloud server and RSUs, which means that the computation complexity of OBUs is independent of the number of attributes.

The remainder of this paper is organized as follows. The related work is overviewed in Section 2, and technical preliminaries are provided in Section 3. The system framework, security model and system definition are provided in Section 4, and our construction of the proposed scheme is elaborated in Section 5. The security and performance analyses are described in Section 6 and Section 7. The conclusions are given in Section 8.

## 2. Related Works

Over recent years, eavesdropping on messages, tampering with messages and forging warning messages by malicious attackers are security threats in VCC, and many related works have been proposed that have concentrated on confidentiality, access control, authentication, etc.

Pietrowicz et al. [12] adopted identity based encryption (IBE) algorithms to effectively address the challenges in providing secure communications in vehicle networks. Mallissery et al. [13] adopted the RSU geolocation key to encrypt the exchanged messages in a vehicular ad-hoc network (VANET), which provides location confidentiality against vehicles outside the zone. The weakness is that this scheme limits the scope of message sharing only to one RSU. Nema et al. [14] proposed an RSA-algorithm-based encryption and decryption approach to provide message confidentiality in VANETs. However, all of the above schemes do not consider the fine-grained access control of the transmitted message.

ABE, introduced by Sahai and Waters, is cryptographic technique to implement fine-grained access control for encrypted messages [15,16]. In fact, ABE can be adopted in many applications to realize message confidentiality and access control in vehicular communication [17,18,19,20]. Huang et al. [17] proposed a security policy enforcement scheme to achieve secure message dissemination, which is the first one to introduce CP-ABE in VANET. The main drawback of this scheme is that the vehicles under different secure groups of RSUs cannot share messages with each other directly, which was improved in [18]. For emergency services, Yeh et al. [19] proposed an access control scheme in VANETs to send messages to nearby rescue vehicles securely with ABE. Xia et al. [9] divided the attributes of vehicles into two types, dynamic attributes and persistent attributes. Dynamic attribute values would change frequently, while persistent attributes such as police car and sprinkler would never change. This brings new challenges with respect to the heavy key management of AA, since it must re-generate secret keys for both persistent attributes and dynamic attributes when any dynamic attribute changes. To solve the issue of heavy key management by adopting ABE in VCC, Liu et al. [20] extended the CP-ABE algorithm with hierarchical authorities, which can reduce the key management of a single center authority. Nevertheless, none of the above ABE-based schemes can provide mechanisms to authenticate vehicles before handling the messages.

Message authentication of vehicles, which determines that a message is from a valid source, is another important security issue in vehicular communication networks. In consideration of the identity privacy of vehicles, the traditional IBS method is no longer applicable [21]. Sánchez-Garcíaby et al. [22] proposed an electronic identity (eID) based secure authentication scheme in VANETs, which can protect drivers’ real identities. The vehicle broadcasts a message containing the certificate signed by eID to prove its identity when receiving the authentication request. Kang et al. [23] integrated pseudonyms with IBS in vehicular communication, which could not only authenticate the messages, but also protect the privacy of the message sender. Chim et al. [24] adopted anonymous credentials to guarantee the identity of driver to be unlinkable to any party. However, in these two anonymous schemes, the vehicle must preset a large number of anonymous keys in order to randomly choose one to sign messages, and the authority or RSU must hold the anonymous certificates of all the vehicles in order to authenticate vehicles, which creates a heavy overhead for key management. Instead of suffering from extra overhead, as in previous anonymous identity-based schemes, ABS is introduced in VCC to ensure anonymous authentication. In order to achieve message verification and maintain anonymity, Liu et al. [20] utilized ABS to enforce message authentication.

However, most existing ABE and ABS schemes introduce heavy computation overheads in the encryption, decryption and signing phases, and these computation costs grow linearly [25,26]. Therefore, OBUs that have limited resources may encounter serious challenges during these processes [27]. To reduce the computational burden of the OBUs of vehicles, Xia et al. [9] introduced an outsourced decryption construction for ABE in VCC, but this scheme requires each RSU to restore secret keys for all vehicles and ignores the high encryption cost of ABE. Liu et al. [28] proposed a secure message dissemination construction for vehicle networks, in which the local decryption computation cost can be outsourced to nearest RSU, but this scheme ignores the computation cost of message encryption with ABE. Ma et al. [29] proposed two CP-ABE based mechanisms for achieving both outsourced encryption and outsourced decryption. However, this scheme is not practical in VCC.

## 3. Technical Preliminaries

### 3.1. Bilinear Map

Let $G_{0}$ and $G_{T}$ be two multiplicative groups with the same prime order *p*. A map $e:G_{0}\times G_{0}\to G_{T}$ with the following properties is said to be bilinear:

(1) Computability. There is a polynomial time algorithm to compute $e(g,h)\in G_{T}$ for any $g,h\in G_{0}$.
(2) Bilinearity. For all $g,h\in G_{0}$ and $a,b\in {\mathbb{Z}}_{p}$, we have $e(g^{a},h^{b})=e{\left(g,h\right)}^{ab}$.
(3) Non-degeneracy. There exists $g,h\in G_{0}$ such that e(g,h)≠1.

### 3.2. Access Tree

Let *T* be a tree representing an access policy. Each non-leaf node *x* of the tree represents a threshold gate. Let *num_x_* denote the number of children of a node *x*, and *k_x_* represent its threshold value, then 1 ≤ *k_x_* ≤ *num_x_*. For each leaf node *x* of the tree, we have *k_x_* = 1, and denote *attr_x_* as an attribute associated with it. For a non-leaf node *x*, the child nodes of *x* are numbered from 1 to *num_x_*. The function *parent*(*x*) represents a parent node of the node *x*, *index*(*x*) returns the index value of node *x*.

We let $T_{x}$ be the sub-tree rooted at node *x* in *T*. We denote the result as $T_{x}(r)=1$ if the attribute set *r* satisfies the access tree $T_{x}$. Then the value of $T_{x}(r)$ is computed in the following. If *x* is a leaf node and $attr_{x}\in r$, $T_{x}(r)$ returns 1. If *x* is a non-leaf node, we compute $T_{n}(r)$ for all children n of node *x*. If at least *k_x_* children return 1, $T_{x}(r)$ returns 1.

### 3.3. Ciphertext-Policy, Attribute-Based Encryption

In a typical CP-ABE system, the access policy is expressed as a tree over a set of attributes. The CP-ABE scheme is composed of the following four algorithms.

(1) *Setup*($1^{\lambda }$): On input of a security parameter λ, the algorithm outputs a public key *PK* and a master key *MK*.
(2) *KeyGen*(*MK*, *PK*, *S*): On input of the master key *MK*, public key *PK* and a set *S* of attributes, the algorithm outputs a secret key *SK*.
(3) *Enc*(*PK*, *M*, *T_a_*): On input of the public key *PK*, a message *M* and an access policy *T_a_*, the algorithm outputs a ciphertext *CT*.
(4) *Dec*(*PK*, *CT*, *SK*): On input of the public key *PK*, a ciphertext *CT* associated with an access policy *T_a_* and a secret key *SK*, the algorithm outputs the message *M* if $S\in T_{a}$.

### 3.4. Attribute-Based Signature

An ABS scheme that provides anonymous message authentication generally consists of the following four algorithms.

(1) *Setup*($1^{\lambda }$). On input of a security parameter λ, AA generates the public key *PK* and master key *MK*.
(2) *KeyGen*(*MK*, *PK*, *S*). On input of the master key *MK*, public key *PK*, and a set of attributes *S*, AA generates the secret key *SK*.
(3) *Sign*(*PK*, *SK*, *M*, *T_c_*). On input of the public key *PK*, a secret key *SK* of signer, a message *M* and a predicate policy *T_c_*, the signer generates a signature *ST* for *M*.
(4) *Verify*(*PK*, *M*, *T_c_*, *ST*). On input of the public key *PK*, a message *M*, a predicate policy *T_c_* and a signature *ST*, the verifier checks *ST*. If the signer’s attributes satisfy *T_c_*, it outputs true.

## 4. System Overview

### 4.1. System Framework

**Figure 1:**
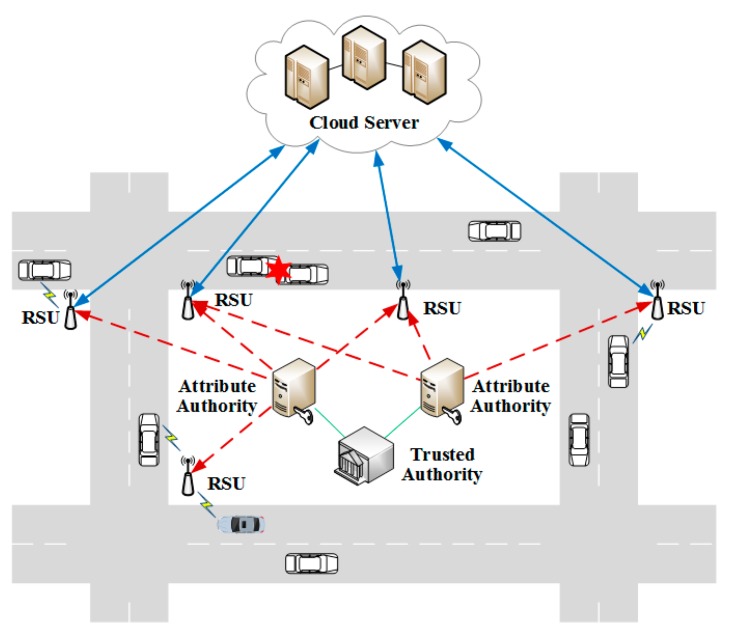
System framework of SmartVeh.

The system framework of SmartVeh consists of the following parties: TA, AA, cloud server, RSUs and vehicles, as shown in Figure 1. The TA is viewed as a fully trusted party that takes charge of managing AAs and generating system parameters and secret parameter to AAs. The AAs are also trusted and independent of each other. According to the different types of attributes managed by the AA, persistent AA is responsible for generating the persistent attributes of vehicles, and dynamic AA is responsible for generating the dynamic attributes of vehicles. A semi-trusted cloud server which has powerful computation and storage capabilities is intended to perform the outsourced encryption and signing computations. The RSUs are interconnected through wired lines, and provide wireless connections to vehicles. We assume that there are the dense of RSUs deployed near the road in the city, and the RSUs are responsible for performing access control with vehicles, and authenticating the origin of messages by verifying the signature of vehicles. If the signature verification is passed, RSUs would partially decrypt the encrypted messages, and then broadcast them to vehicles. The vehicles with OBUs and powerful sensors are a set of nodes that are moving on the road, and communicate with each other through RSUs. When a vehicle communicates with others, it encrypts the message with an access policy and signs message with its attributes before broadcasting to others, and intended receivers can decrypt the ciphertext with their attributes.

### 4.2. Security Model

In this work, we consider TA and AA to be trusted, while the cloud server and RSUs are honest but curious. It means they may learn sensitive information from the broadcast message. Specifically, the security requirements are defined as follows:

(1) Message confidentiality. The messages should be transmitted in encrypted form, and the vehicles which cannot satisfy the access policy defined by the message sender should not be allowed to access the plaintext of the message. Meanwhile, the cloud server and RSUs cannot recover the broadcast message.
(2) Fine-grained access control. The vehicle can enforce an access policy for each broadcast message, which designates the messages that the vehicle is allowed to access.
(3) Message authentication. If message sender’s attributes could not satisfy the predicate policy, the message broadcast should not succeed.
(4) Collusion resistance. The message access should not be successful if either of the vehicles cannot satisfy the access policy alone. Further, even if unauthorized vehicles collude with the RSU, the access should not take effect.

### 4.3. System Definition

According to the SmartVeh framework, our scheme consists of these ten algorithms.

(1) $Setup(1^{\lambda })$: On input of a security parameter λ, the TA outputs a system public key *PK* and a master key *MK*.
(2) CreateAA(PK,MK,A): On input of *PK* and *MK*, a set of attributes A of AA, the TA outputs the master secret key *MSK* for AA.
(3) $KeyGen(PK,MSK,S_{i})$: On input of *PK* and *MSK*, a set of managed attributes $S_{i}$ of the vehicle, the AA outputs the secret key $SK_{i}$ for each vehicle.
(4) $Cloud.Encrypt(PK,{\left\{T_{a}^{\left(i\right)}\right\}}_{i=1}^{2})$: On input of *PK*, access policies ${\left\{T_{a}^{\left(i\right)}\right\}}_{i=1}^{2}$ in different AAs, the cloud server outputs a partially encrypted ciphertext $CT^{\prime }$.
(5) $Vehicle.Encrypt(PK,M,CT^{\prime })$: On input of *PK*, a message *M* and a partial ciphertext $CT^{\prime }$, the vehicle outputs a ciphertext CT.
(6) $Cloud.Sign(CT,T_{c},SK_{i}^{\prime })$: On input of a ciphertext *CT*, a predicate policy $T_{c}$ and an outsourced secret key $SK_{i}^{\prime }$ which is a part of secret key, the cloud server outputs a signing token *SN* and a partial signature $ST^{\prime }$.
(7) $Vehicle.Sign(ST^{\prime },SK)$: Given a partial signature $ST^{\prime }$ and secret key *SK*, the vehicle generates a thorough signature *ST*.
(8) $Verify(ST,SN,T_{c})$: On input of a signature *ST*, a signing token *SN* and a predicate policy $T_{c}$, the RSU outputs true if the sender vehicle’s attributes satisfy $T_{c}$.
(9) $RSU.Decrypt(PK,SK_{i}^{\prime \prime },CT)$: On input of *PK*, a ciphertext *CT*, a outsourced secret key $SK_{i}^{\prime \prime }$ which is also a part of secret key, the RSU outputs a partially decrypted ciphertext $CT_{p}$ if the attribute set satisfies the access policy.
(10) $Vehicle.Decrypt(CT_{p},SK_{i})$: The vehicle takes a $CT_{p}$ and a secret key $SK_{i}$ as input, and outputs the plaintext *M*.

## 5. Construction of SmartVeh

In order to achieve secure message broadcasting, we provided an access control framework for encrypted messages in VCC by employing a delegation mechanism based on HABE, and utilized ABS to enforce message authentication, which can authenticate messages by verifying that the sender’s attributes satisfy $T_{c}$ in the ciphertext.

### 5.1. System Setup

The TA first runs the Setup algorithm to choose two multiplicative groups with prime order *p*, that are $G_{0}$ and $G_{T}$, and a bilinear map $e:G_{0}\times G_{0}\to G_{T}$. Then, the TA randomly chooses $g,h\in G_{0}$ and $\alpha ,\beta \in {\mathbb{Z}}_{p}$, and chooses cryptographic hash functions $H_{1}:\{0,1\}*\to {\mathbb{Z}}_{p}^{*}$, $H_{2}:\{0,1\}*\to$$G_{0}$. Finally, the TA outputs a system public key $PK=(g,g^{\alpha },g^{\beta },h,h^{\beta },e{\left(g,g\right)}^{\alpha \beta })$ and a master key MK=(α,β).

### 5.2. Authority Setup

Our scheme divides the attributes of vehicle into two types, persistent attributes and dynamic attributes, which are managed by different AAs independently. The TA runs the CreateAA algorithm to select a random but unique value $\nu_{i}\in {\mathbb{Z}}_{p}$ for AA*_i_*. For the attribute set A managed by AA*_i_*, the TA chooses random $r_{i,j}$ for each attribute in it. Then the TA computes the master secret key for AA*_i_* as

(1) $$MSK_{i}=(D_{i}^{\prime }=g^{(\alpha +\nu_{i})\beta },D_{i,1}^{\prime }=g^{\nu_{i}},{\left\{{\bar{D}}_{i,j}=g^{\nu_{i}\beta }H_{1}{\left(j\right)}^{r_{i,j}},{\bar{D}}_{i,j}^{\prime }=g^{r_{i,j}}\right\}}_{j\in A})$$

### 5.3. Key Generation

For each vehicle, the AA*_i_* runs the KeyGen algorithm to choose a unique secret $\gamma_{i}\in {\mathbb{Z}}_{p}$ and a random $\varepsilon_{i}\in {\mathbb{Z}}_{p}$. For each attribute j in the attribute set $S_{i}$ of vehicle in AA*_i_*, the AA*_i_* chooses a random $u_{i,j}\in {\mathbb{Z}}_{p}$. Finally, AA*_i_* outputs the key as

(2) $$AK_{i}=({\left\{{\tilde{D}}_{i,j}={\bar{D}}_{i,j}\cdot g^{\gamma_{i}\beta }H_{1}{\left(j\right)}^{u_{i,j}}=g^{(\nu_{i}+\gamma_{i})\beta }H_{1}{\left(j\right)}^{r_{i,j}+u_{i,j}},{\bar{D}}_{i,j}^{\prime }={\bar{D}}_{i,j}^{\prime }\cdot g^{u_{i,j}}=g^{r_{i,j}+u_{i,j}}\right\}}_{j\in S})$$

Thus the vehicle’s secret key in AA*_i_* is:

(3) $$SK_{i}=(D_{i}=D_{i}^{\prime }\cdot g^{\gamma_{i}\beta }=g^{(\alpha +\nu_{i}+\gamma_{i})\beta },D_{i,1}=D_{i,1}^{\prime }\cdot g^{\gamma_{i}}h^{\varepsilon_{i}}=g^{\nu_{i}+\gamma_{i}}h^{\varepsilon_{i}},D_{i,2}=g^{\varepsilon_{i}},AK_{i})$$

For example, an ambulance can get secret keys for vehicle type from the AA_1_ for persistent attributes, and get secret keys for road and direction from the AA_2_ for dynamic attributes.

### 5.4. Message Broadcasting

Before broadcasting the message to the RSUs, the vehicle first selects a symmetric key $DK\in {\mathbb{Z}}_{p}$ randomly. Then the vehicle encrypts *M* by utilizing a symmetric encryption algorithm, and the result is outputted as $C=SE_{DK}(M)$. Then the vehicle defines a collection of access policies ${\left\{T_{a}^{\left(i\right)}\right\}}_{i=1}^{2}$, where $T_{a}^{\left(i\right)}$ is the access tree in AA*_i_*, such as “police car OR ambulance”, “(normal road AND east) AND (eall road AND north)”.

#### 5.4.1. Cloud Encryption

The cloud server runs the Cloud.Encrypt algorithm to execute outsourcing encryption. First, the cloud server chooses a polynomial $p_{x}$ for each node x in $T_{a}^{\left(i\right)}$. The polynomials are selected in a top-down manner. For each node *x* in $T_{a}^{\left(i\right)}$, the cloud server sets the degree $d_{x}$ of $p_{x}$ to be $k_{x}-1$.

The algorithm selects a random $s_{i}\in {\mathbb{Z}}_{p}$ and sets $p_{R}(0)=s_{i}$ for the root node R. Then the algorithm chooses $d_{R}$ other points of $p_{R}$ randomly to complete the definition. For the other node *x*, the algorithm sets $p_{x}(0)=p_{parent(x)}(index(x))$ and chooses $d_{x}$ other points randomly to complete the definition. In $T_{a}^{\left(i\right)}$, let *Y_i_* be the set of leaf nodes. Then, the cloud server returns the result as

(4) $$CT_{i}=(T_{a}^{\left(i\right)},{\left\{{\tilde{C}}_{i,y}=g^{p_{y}(0)},{\tilde{C}}_{i,y}^{\prime }=H_{1}{\left(attr_{y}\right)}^{p_{y}(0)}\right\}}_{y\in Y_{i}})$$

Finally, the cloud server outputs a partial ciphertext $CT^{\prime }$ as

(5) $$CT^{\prime }=({\left\{C_{i,3}^{\prime }=g^{\beta s_{i}},C_{i,4}^{\prime }=h^{\beta s_{i}},CT_{i}\right\}}_{i\in \{1,2\}})$$

#### 5.4.2. Vehicle Encryption

With the partial ciphertext $CT^{\prime }$, the vehicle runs the Vehicle.Encrypt algorithm to randomly choose $t\in {\mathbb{Z}}_{p}$, compute $C_{1}=DK\cdot e{\left(g,g\right)}^{\alpha \beta t}$ and $C_{2}=g^{t}$. Then, the vehicle computes $C_{i,3}=C_{i,3}^{\prime }\cdot g^{\beta t},C_{i,4}=C_{i,4}^{\prime }\cdot h^{\beta t}$ and outputs the ciphertext CT as

(6) $$CT=(C=SE_{DK}(M),C_{1}=DK\cdot e{\left(g,g\right)}^{\alpha \beta t},C_{2}=g^{t},{\left\{C_{i,3}=g^{\beta (s_{i}+t)},C_{i,4}=h^{\beta (s_{i}+t)},CT_{i}\right\}}_{i\in \{1,2\}})$$

#### 5.4.3. Cloud Signing

The encrypted messages must be authenticated, since the messages may be forged by attackers. Then the vehicle computes $S_{0}=H_{2}(CT)$, and sends the ciphertext *CT*, a predicate policy $T_{c}$, such as “(middle road AND east) AND location of accident”, an outsourced secret key $SK_{k}^{\prime }=\{AK_{k}\}$ corresponding to attribute set $S_{k}$ in AA to the cloud server through RSUs. The cloud server runs the Cloud.Sign algorithm to execute computation outsourcing. For each node *x* of predicate policy $T_{c}$, the cloud server chooses polynomial $q_{x}$ in a top-down manner, and sets the degree $d_{x}^{\prime }$ of $q_{x}$ to be $k_{x}^{\prime }-1$.

Starting from *R*, the algorithm first selects a random $r\in {\mathbb{Z}}_{p}$ and sets $q_{R}(0)=r$. Then, the algorithm randomly chooses $d_{R}^{\prime }$ other points of $q_{R}$ to complete the definition. For the other node *x*, it sets $q_{x}(0)=q_{parent(x)}(index(x))$ and then selects $d_{x}^{\prime }$ other points randomly to define $q_{x}$ completely.

In $T_{c}$, let *Z* be the set of leaf nodes. Then, the cloud server outputs the signing token *SN* as

(7) $$SN={\left\{{\tilde{K}}_{z}=g^{q_{z}(0)},{\tilde{K}}_{z}^{\prime }=H_{1}{\left(attr_{z}\right)}^{q_{z}(0)}\right\}}_{z\in Z}$$

The cloud server randomly chooses $t_{j}\in {\mathbb{Z}}_{p}$ for each node j∈Z, and computes with $SK_{k}^{\prime }$ as follows.

(1) If $j\in S_{k}\cap Z$, the cloud server computes ${\tilde{S}}_{j}={\left({\tilde{D}}_{k,j}\cdot H_{1}{\left(j\right)}^{t_{j}}\right)}^{1/r}=g^{(\nu_{k}+\gamma_{k})\beta /r}\cdot H_{1}{\left(j\right)}^{\left(r_{k,j}+u_{k,j}+t_{j}\right)/r}$, and ${\tilde{S}}_{j}^{\prime }={\left({\tilde{D}}_{k,j}^{\prime }\cdot g^{t_{j}}\right)}^{1/r}=g^{\left(r_{k,j}+u_{k,j}+t_{j}\right)/r}$.
(2) If $j\in Z/S_{k}\cap Z$, the cloud server computes ${\tilde{S}}_{j}={\left(H_{1}{\left(j\right)}^{t_{j}}\right)}^{1/r}=H_{1}{\left(j\right)}^{t_{j}/r}$, and ${\tilde{S}}_{j}^{\prime }={\left(g^{t_{j}}\right)}^{1/r}=g^{t_{j}/r}$.

Finally, the cloud server randomly selects $\lambda \in {\mathbb{Z}}_{p}$ and outputs the partial signature $ST^{\prime }$ as

(8) $$ST^{\prime }=(S_{1}^{\prime }=H_{2}{\left(CT\right)}^{\lambda },S_{2}^{\prime }=g^{\lambda },S_{3}={\left\{{\tilde{S}}_{j},{\tilde{S}}_{j}^{\prime }\right\}}_{j\in Z})$$

#### 5.4.4. Vehicle Signing

With the partial signature generated by the cloud server, the vehicle first runs the Vehicle.Sign algorithm to randomly choose $\mu \in {\mathbb{Z}}_{p}$ and compute $S_{1}=S_{1}^{\prime }\cdot {\left(S_{0}\right)}^{\mu }\cdot D_{k}$ and $S_{2}=S_{2}^{\prime }\cdot g^{\mu }$. At last, the vehicle generates the encrypted message’s signature ST as

(9) $$ST=(S_{1}=H_{2}{\left(CT\right)}^{\lambda +\mu }\cdot g^{(\alpha +\nu_{k}+\gamma_{k})\beta },S_{2}=g^{\lambda +\mu },S_{3})$$

The vehicle sends the signature *ST* with encrypted message to the connected RSUs, and the message will be broadcasted to other vehicles.

### 5.5. Message Decryption

When receiving the encrypted and signed message, the recipient RSU runs the Verify algorithm to verify that the message is from an authorized source.

#### 5.5.1. RSU Verifying

The RSU runs the *VerNode* algorithm, which takes as input *ST*, *SN* and a node *x* of $T_{c}$.

(1) If *x* is a leaf node, then we set $w=attr_{x}$. If w∈S∩Z, then
  (10) $$VerNode(ST,SN,x)=\frac{e({\tilde{S}}_{w},{\tilde{K}}_{x})}{e({\tilde{S}}_{w}^{\prime },{\tilde{K}}_{x}^{\prime })}=\frac{e(g^{(\nu_{k}+\gamma_{k})\beta /r}H_{1}{\left(z\right)}^{\left(r_{k,w}+u_{k,w}+t_{w}\right)/r},g^{q_{x}(0)})}{e(g^{\left(r_{k,w}+u_{k,w}+t_{w}\right)/r},H_{1}{\left(attr_{x}\right)}^{q_{x}(0)})}=e{\left(g,g\right)}^{(\nu_{k}+\gamma_{k})\beta /r\cdot q_{x}(0)}$$
  If w∈Z/S∩Z, then
  (11) $$VerNode(ST,SN,x)=\frac{e({\tilde{S}}_{w},{\tilde{K}}_{x})}{e({\tilde{S}}_{w}^{\prime },{\tilde{K}}_{x}^{\prime })}=\frac{e(H_{1}{\left(w\right)}^{t_{w}/r},g^{q_{x}(0)})}{e(g^{t_{w}/r},H_{1}{\left(attr_{x}\right)}^{q_{x}(0)})}=1$$
(2) If *x* is a non-leaf node, the algorithm VerNode(ST,SN,x) computes as follows. It calls the VerNode(ST,SN,n) algorithm for each child node *n* of *x*, and outputs the result as $I_{n}$.

We denote $S_{x}$ as an arbitrary $k_{x}$-sized set of child nodes n such that $I_{n}\neq ⊥$. If no such set exists, it returns ⊥. Otherwise, the algorithm computes the $I_{x}$.

(12) $$\begin{array}{cc} I_{x} & =\prod_{n\in S_{x}}I_{n}{}^{\Delta_{j,S_{x}^{\prime }}(0)}=\prod_{n\in S_{x}}{\left(e{\left(g,g\right)}^{(\nu_{k}+\gamma_{k})\beta /r\cdot q_{parent(n)}(index(n))}\right)}^{\Delta_{j,S_{x}^{\prime }}(0)}=\prod_{n\in S_{x}}e{\left(g,g\right)}^{(\nu_{k}+\gamma_{k})\beta /r\cdot q_{x}(j)\cdot }{}^{\Delta_{j,S_{x}^{\prime }}(0)}\\ & =e{\left(g,g\right)}^{(\nu_{k}+\gamma_{k})\beta /r\cdot q_{x}(0)} \end{array}$$

where j=index(n) and $S_{x}^{\prime }=\{index(n):n\in S_{x}\}$. Then, we can define the evaluation result for predicate tree $T_{c}$ as *I*, if $T_{c}$ is satisfied.

(13) $$I=VerNode(ST,SN,R)=e{\left(g,g\right)}^{(\nu_{k}+\gamma_{k})\beta /r\cdot q_{R}(0)}=e{\left(g,g\right)}^{(\nu_{k}+\gamma_{k})\beta /r\cdot r}=e{\left(g,g\right)}^{(\nu_{k}+\gamma_{k})\beta }$$

Finally, the RSU checks whether the equation holds.

(14) $$\frac{e(g,S_{1})}{e(H_{2}(CT),S_{2})\cdot I}=\frac{e(g,H_{2}{\left(CT\right)}^{\lambda +\mu }\cdot g^{(\alpha +\nu_{k}+\gamma_{k})\beta })}{e(H_{2}(CT),g^{\lambda +\mu })\cdot e{\left(g,g\right)}^{(\nu_{k}+\gamma_{k})\beta }}=e{\left(g,g\right)}^{\alpha \beta }$$

If the equation holds, then RSU accepts *ST* and partially decrypts the encrypted message for vehicles that satisfy the access policy.

#### 5.5.2. RSU Decryption

With part of the secret key $SK_{k}^{\prime \prime }=(D_{k,1},D_{k,2},AK_{k})$ from the vehicle corresponding to attribute set $S_{k}$, the RSU runs the RSU.Decrypt algorithm to decrypt the *CT*. In order to evaluate whether the vehicle’s attributes satisfy $T_{a}^{\left(k\right)}$ or not, the RSU runs the *DecNode* algorithm, which takes as input $CT_{k}$, $SK_{k}^{\prime \prime }$, and a node *x* from $T_{a}^{\left(k\right)}$.

(1) If *x* is a leaf node, then we let $w=attr_{x}$ and compute the following. If $w\in S_{k}$, then
  (15) $$DecNode(CT_{k},SK_{k}^{\prime \prime },x)=\frac{e({\tilde{D}}_{k,w},{\tilde{C}}_{k,x})}{e({\tilde{D}}_{k,w}^{\prime },{\tilde{C}}_{k,x}^{\prime })}=\frac{e(g^{(\nu_{k}+\gamma_{k})\beta }H_{1}{\left(w\right)}^{r_{k,w}+u_{k,w}},g^{p_{x}(0)})}{e(g^{r_{k,w}+u_{k,w}},H_{1}{\left(attr_{x}\right)}^{p_{x}(0)})}=e{\left(g,g\right)}^{\left(\nu_{k}+\gamma_{k})\beta p_{x}(0\right)}$$
  If $z\notin S_{k}$, then $DecNode(CT_{k},SK_{k}^{\prime \prime },x)=⊥$.
(2) If *x* is a non-leaf node, the algorithm $DecNode(CT_{k},SK_{k}^{\prime \prime },x)$ computes the following. It calls $DecNode(CT_{k},SK_{k}^{\prime \prime },n)$ for each child node *n* of *x*, and generates the result as $F_{k,n}$. Let $S_{x}$ be an arbitrary $k_{x}$-sized set of child nodes n such that $F_{k,n}\neq ⊥$. Similar to the verifying process, the algorithm computes as follows.
  (16) $$\begin{array}{cc} F_{k,x} & =\prod_{n\in S_{x}}F_{k,n}{}^{\Delta_{j,S_{x}^{\prime }}(0)}=\prod_{n\in S_{x}}{\left(e{\left(g,g\right)}^{\left(\nu_{k}+\gamma_{k})\beta \cdot p_{parent(n)}(index(n)\right)}\right)}^{\Delta_{j,S_{x}^{\prime }}(0)}=\prod_{n\in S_{x}}e{\left(g,g\right)}^{(\nu_{k}+\gamma_{k})\beta \cdot p_{x}(j)\cdot }{}^{\Delta_{j,S_{x}^{\prime }}(0)}\\ & =e{\left(g,g\right)}^{\left(\nu_{k}+\gamma_{k})\beta \cdot p_{x}(0\right)} \end{array}$$

If the receiver owns enough attributes to satisfy $T_{a}^{\left(k\right)}$, we set the evaluation result as $F_{k}$.

(17) $$F_{k}=DecNode(CT_{k},SK_{k}^{\prime \prime },R)=e{\left(g,g\right)}^{\left(\nu_{k}+\gamma_{k})\beta p_{R}(0\right)}=e{\left(g,g\right)}^{(\nu_{k}+\gamma_{k})\beta s_{k}}$$

RSU computes

(18) $$B_{k}=\frac{e(D_{k,1},C_{k,3})}{e(D_{k,2},C_{k,4})}=\frac{e(g^{\nu_{k}+\gamma_{k}}h^{\varepsilon_{k}},g^{\beta (s_{k}+t)})}{e(g^{\varepsilon_{k}},h^{\beta (s_{k}+t)})}=e{\left(g,g\right)}^{\left(\nu_{k}+\gamma_{k})\beta (s_{k}+t\right)}$$

and

(19) $$A_{k}=B_{k}/F_{k}=e{\left(g,g\right)}^{\left(\nu_{k}+\gamma_{k})\beta (s_{k}+t\right)}/e{\left(g,g\right)}^{(\nu_{k}+\gamma_{k})\beta s_{k}}=e{\left(g,g\right)}^{(\nu_{k}+\gamma_{k})\beta t}$$

Hence, if the vehicle’s attributes satisfy $T_{a}^{\left(k\right)}$, the RSU sends the result $CT_{p}=(C,C_{1},C_{2},A_{k})$ to the vehicle.

#### 5.5.3. Vehicle Decryption

After receiving the result from the RSU, the vehicle runs the Vehicle.Decrypt algorithm to recover *DK* with its own secret key.

(20) $$DK=\frac{C_{1}\cdot A_{k}}{e(C_{2},D_{k})}=\frac{DK\cdot e{\left(g,g\right)}^{\alpha \beta t}\cdot e{\left(g,g\right)}^{(\nu_{k}+\gamma_{k})\beta t}}{e(g^{t},g^{(\alpha +\nu_{k}+\gamma_{k})\beta })}=\frac{DK\cdot e{\left(g,g\right)}^{\alpha \beta t}}{e(g^{t},g^{\alpha \beta })}$$

Finally, the vehicle can recover the message *M* with *DK* based on the symmetric decryption algorithm, while the unauthorized vehicles are prevented from accessing it.

## 6. Security Analysis

The construction of SmartVeh is based on CP-ABE [25] and ABS [26], which have been proved secure, thus our scheme has the same security property as these. Then we discuss the security properties of SmartVeh, which not only provides message confidentiality, but also guarantees fine-grained access control, efficient message authentication and collusion resistance.

### 6.1. Message Confidentiality

The broadcast message in our scheme is first encrypted with a symmetric encryption technique. Then the *DK* is encapsulated by access policy. Hence, message confidentiality against outside vehicles which do not have enough attributes can be guaranteed. In the message broadcasting phase, the cloud server executes most of encryption computations for the vehicle. However, the cloud server cannot access the plaintext of message without the secret key. Moreover, if the attribute set of the vehicle cannot satisfy the $T_{a}$ in the ciphertext, the value $A_{k}=e{\left(g,g\right)}^{(\nu_{k}+\gamma_{k})\beta t}$ cannot be computed by the RSUs to get *DK* in the message decryption phase. Therefore, only vehicles that satisfy *T_a_* can decrypt the encrypted message, and message confidentiality against a semi-trusted cloud server and RSUs is also guaranteed.

### 6.2. Fine-Grained Access Control

Our work used the CP-ABE mechanism to protect *DK*, and ensure flexibility by specifying the access policies of vehicles. In the message encryption phase, the sender is able to protect the symmetric key with an expressive access policy, and broadcast the encrypted message through RSUs. Specifically, the access policy in the ciphertext can be represented by flexible access tree. In this way, our scheme can dramatically increase the flexibility and represent any desired access conditions.

### 6.3. Message Authentication

In our work, the ABS technique was adopted to achieve message authentication with privacy preservation. The adversary, such as a malicious vehicle, may want to forge a signature with an unsatisfied predicate policy, so that fake messages have a reliable source. However, as proved in [26], our work is secure under the computational Diffie-Hellman assumption, since the adversary cannot forge a valid *ST* with a non-negligible probability.

### 6.4. Collusion Resistance

Malicious vehicles may collude to combine their secret keys to decrypt a ciphertext that each of them cannot access individually. However, the secret key outputted by AA in our scheme is generated with random $\gamma_{i}$, which is unique for each vehicle. Thus, even if two or more vehicles combine their attributes to satisfy the access policies, the value $F_{k}=e{\left(g,g\right)}^{(\nu_{k}+\gamma_{k})\beta s_{k}}$ cannot be computed. Moreover, even if malicious vehicles collude with RSUs to decrypt the encrypted message, the collusion will not succeed.

## 7. Performance Analysis

### 7.1. Functionality Comparisons

In this part, we will analyze the performance of several ABE-based message sharing schemes. The results are shown in Table 1. The functionality comparison of our scheme with these schemes in VCC is in terms of message confidentiality, hierarchical authorities, persistent attribute key generation, anonymous authentication and computation outsourcing.

First, the compared schemes all adopt the ABE technique to grant fine-grained access control for vehicular messages. Moreover, only Xia et al. [9], Liu et al. [20] and our scheme clearly define the attributes of vehicles that include persistent attributes and dynamic attributes. However, a persistent attribute key is generated only once in Liu et al. [20] and our scheme, while in Xia et al. [9] it needs to be generated when the vehicles move into another RSU. Further, we can see that in our scheme, Xia et al. [9] and Liu et al. [28] achieve decryption outsourcing, which incur less computation costs for message decryption for resource-limited OBUs in vehicles. This is because the RSU helps the OBU to decrypt the ciphertext. However, the origin of the message is not authenticated in Xia et al. [9] and Liu et al. [28], which may bring security concerns, such as forged messages and man-in-the-middle attacks. Chim et al. [24] and Liu et al. [20] adopt IBS with pseudonym and ABS, respectively, to achieve anonymous authentication, but the pseudonym method creates large extra storage overheads and the standard ABS method would bring large computation costs.

Compared to these schemes, our scheme first introduces HABE to reduce the overhead for key management on a single TA by dividing dynamic and persistent attributes managed by different AAs, which also resolves the problem of single point failure to a certain extent, and the complexity of operations of AAs in the key generation phase is independent of the number of vehicles, which means that our scheme is scalable enough to handle a case where the number of authorized vehicles increases dynamically. Further, our scheme proposes an outsourced architecture to satisfy the lightweight demand of resource-limited OBUs in VCC.

### 7.2. Performance Analysis

We discuss the efficiency of our scheme in terms of message encryption, decryption and signing, and compare the results with Liu et al. [28], Xia et al. [9] and Liu et al. [20], which are related schemes in a vehicular network. Table 2 shows the comparison results. Let $T_{r}$, $T_{0}$, $T_{t}$, $N_{c}$, $N_{u}$ and $N_{d}$ denote the computation cost of the pairing operation, the computation cost of the exponentiation operation in $G_{0}$, the computation cost of the exponentiation operation in $G_{T}$, the number of attributes in the ciphertext, the total number of attributes of the vehicle, and the number of dynamic attributes, respectively. The symmetric encryption and decryption, hash and simple multiplication operations are ignored.

First, we analyzed the computation cost in the key generation phase. As vehicles are moved through different RSUs dynamically along with time, the secret keys should be generated for vehicles by TA. Xia et al. [9] and Liu et al. [28] both need to perform $(3+N_{u})T_{0}$ to generate all secret keys for vehicles. Our scheme and Liu et al. [20] both divide attributes into two types, namely persistent attributes and dynamic attributes. The AA only needs to generate secret keys according to dynamic attributes for vehicles since the value of persistent attributes are not changed. From the table, we can notice that the computation cost of our scheme in this phase is less than that in Liu et al. [20] which needs to generate extra signing keys at the same time.

Second, we discuss the overhead of encryption and decryption of the message. Since Liu et al. [28], Xia et al. [9] and Liu et al. [20] all execute the complex ABE algorithm, the encryption computation costs on the vehicle side of these schemes are $(3N_{c}+1)T_{0}+$$T_{t}$, $(3N_{c}+1)T_{0}+T_{t}$ and $(2N_{c}+1)T_{0}+T_{t}$, respectively, which increase with $N_{c}$. Conversely, the result stay constant in our scheme. For the message decryption phase, the vehicles use secret keys to decrypt the encrypted message recursively in Liu et al. [20], and the computation cost is $(2N_{c}+1)T_{r}+N_{c}T_{t}$. In Liu et al. [28], Xia et al. [9] and our scheme, most of decryption computations are outsourced to nearby RSUs, and the OBUs in vehicles only need one pairing operation to decrypt the partially decrypted message.

In order to analyze the time cost of signing the message, we compared our scheme with Liu et al. [20], which achieves anonymous authentication based on ABS as well, and needs to perform $3N_{u}T_{0}+2T_{t}$ in signing the algorithm, while in our scheme, the cloud server is able to partially sign the ciphertext with a predicate policy and outsourced secret key, which are both sent by the vehicles. The OBUs in the vehicles only need to perform two exponent operations in $G_{0}$. Thus, most of the laborious signing operations in the vehicle are delegated to the cloud server through RSUs, so that the computation overhead of the vehicles can be reduced.

### 7.3. Simulation Evaluation

Next, we analyze the computation cost of our scheme by conducting experiments on a simulated RSU with an Intel CPU at 2.53 GHz and 4 GB RAM. The OBU in the vehicle, which has limited processing power, is simulated by an Android phone with a 1.2 GHz processor [27]. The simulations are developed with a pairing-based cryptography library [30]. A type A elliptic curve of 160-bit group order is chosen. We assume that each vehicle has the same number of persistent attributes and dynamic attributes, which means that each of them has half of the whole attributes.

**Figure 2:**
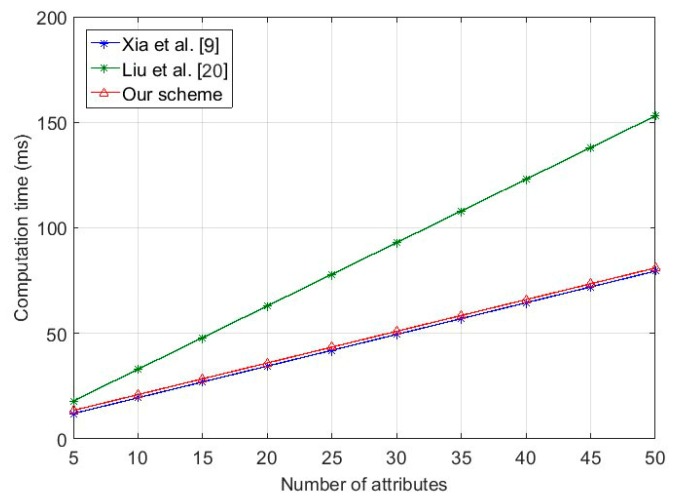
Computation cost of key generation on attribute authority.

From Figure 2, we can observe that the computation costs for key generation in these schemes all grow with $N_{c}$, while those for our scheme and Liu et al. [20] grow at a slower pace than Xia et al. [9], and our scheme costs almost the same as Liu et al. [20].

**Figure 3:**
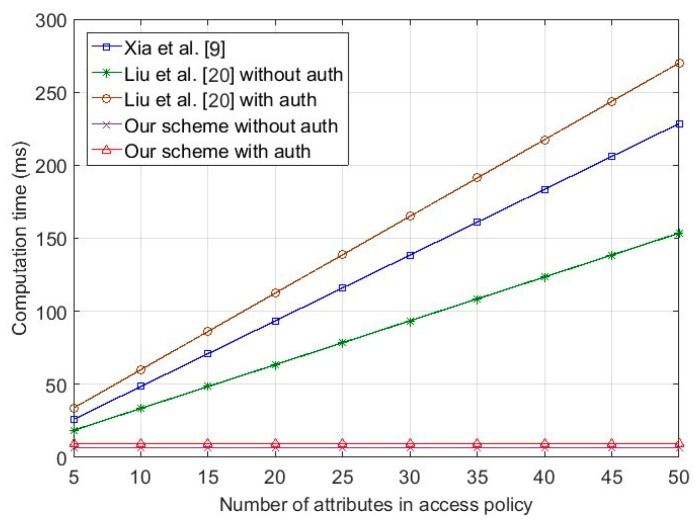
Computation cost of message broadcasting for on-board unit.

**Figure 4:**
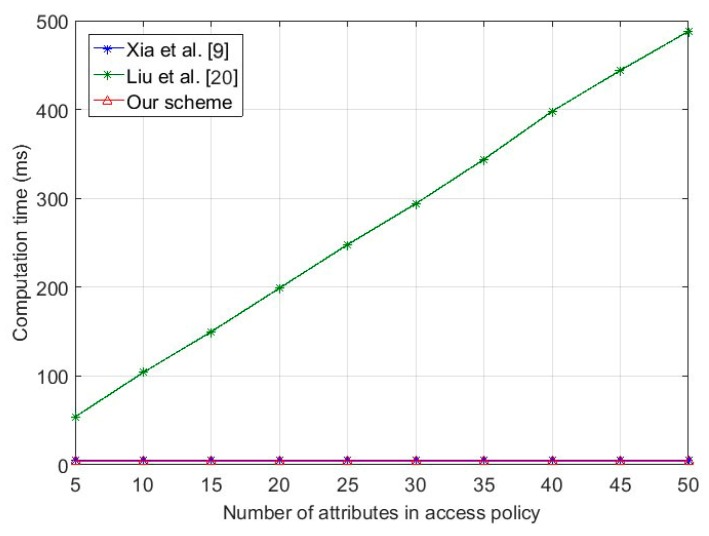
Computation cost of message decryption for on-board unit.

In the message broadcasting phase, the OBU in our scheme encrypts the message with a predefined access policy, and signs the ciphertext. To compare the efficiency of Xia et al. [9], Liu et al. [20] and our scheme, we evaluated the computation costs under two situations, namely non-authentication and authentication. Figure 3 shows that the computation time for message broadcasting is related with $N_{c}$ in *T_a_*. Firstly, the cost of Xia et al. [9] and Liu et al. [20] without authentication increase with $N_{c}$ in *T_a_*, while remaining constant at a low level in our scheme. Then, we compared our scheme with Liu et al. [20] with authentication, to illustrate the encryption efficiency of our authentication scheme. As shown in the figure, the time cost of Liu et al. [20] is related to $N_{c}$ in *T_a_*. Although the results for our scheme are slightly greater than the previous situation, they are still constant, which illustrates that our scheme is more efficient. Figure 4 illustrates the computation time for the OBU by decrypting the ciphertext. The data decryption time of Liu et al. [20] also increased with $N_{c}$ in the *T_a_*, while Xia et al. [9], while, on the contrary, our scheme, based on decryption outsourcing, remained constant.

## 8. Conclusions

This paper proposes a secure and efficient message access control and authentication scheme for VCC based on HABE and ABS. In our scheme, the attributes of vehicle are divided into persistent attributes and dynamic attributes. These two kinds of attributes are managed by different AAs, which reduces the key management for single TAs. To prevent the forging of messages, we adopt ABS to anonymously authenticate the origin of messages in VCC. Considering the resource-limited OBUs in vehicles, our scheme outsources the heavy computations from OBUs to cloud servers and RSUs. The analysis shows that our scheme achieves efficient access control and authentication of messages in VCC.

## Figures and Tables

**Table 1:** Attribute-based message sharing schemes in vehicular cloud computing.

Functions	Yeh et al. [19]	Liu et al. [28]	Chim et al. [24]	Xia et al. [9]	Liu et al. [20]	Our Scheme
Message confidentiality	CP-ABE	CP-ABE	CP-ABE	CP-ABE	HABE	HABE
Hierarchical authorities	No	No	No	No	Yes	Yes
Persistent attribute key generation	-	-	-	Every	Once	Once
Anonymous authentication	No	No	IBS with pseudonym	No	ABS	ABS
Encryption outsourcing	No	No	No	No	No	Yes
Decryption outsourcing	No	Yes	No	Yes	No	Yes
Signing outsourcing	-	-	No	-	No	Yes

**Table 2:** Computation cost.

Schemes	Key Generation (AA)	Message Encryption (OBU)	Message Decryption (OBU)	Message Signing (OBU)
Liu et al. [28]	$(3+N_{u})T_{0}$	$(3N_{c}+1)T_{0}+T_{t}$	$T_{r}$	-
Xia et al. [9]	$(3+N_{u})T_{0}$	$(3N_{c}+1)T_{0}+T_{t}$	$T_{r}$	-
Liu et al. [20]	$(2+4N_{d})T_{0}$	$(2N_{c}+1)T_{0}+T_{t}$	$(2N_{c}+1)T_{p}+N_{c}T_{t}$	$3N_{u}T_{0}+2T_{t}$
Our scheme	$(4+2N_{d})T_{0}$	$3T_{0}+T_{t}$	$T_{r}$	$2T_{0}$
